# Mortality and related risk factors in the co-presentation of tuberculosis and type 2 diabetes mellitus: a population-based study

**DOI:** 10.1080/07853890.2022.2121419

**Published:** 2022-09-16

**Authors:** Po-Tsang Chen, Nai-Cheng Yeh, Shih-Feng Weng, Kai-Jen Tien

**Affiliations:** a Division of Endocrinology and Metabolism, Department of Internal Medicine, Chi Mei Medical Center, Liouying, Taiwan; b Division of Endocrinology and Metabolism, Department of Internal Medicine, Chi Mei Medical Center, Tainan, Taiwan; c Department of Healthcare Administration and Medical Informatics, College of Health Sciences, Kaohsiung Medical University, Kaohsiung, Taiwan; d Center for Medical informatics and Statistics, Office of R&D, Kaohsiung Medical University, Kaohsiung, Taiwan; e Department of Medical Research, Kaohsiung Medical University Hospital, Kaohsiung, Taiwan

**Keywords:** Type 2 diabetes, tuberculosis, mortality, Taiwan

## Abstract

**Objective:**

Patients with type 2 diabetes mellitus (T2DM) are often immunosuppressed and susceptible to infectious diseases. We investigated the mortality and related risk factors of active TB disease in patients with T2DM in Taiwan.

**Materials and Methods:**

The data of 1258 patients diagnosed with both T2DM and active TB disease from January 1 to December 31, 2002 (T2DM–TB group) were retrieved from the Taiwan National Health Insurance Research Database. Patients in the T2DM–TB group were matched by age, sex, and comorbidities to a control group of 10,064 T2DM patients without TB disease (T2DM group). Patients were followed up since TB diagnosis until death or 31 December 2011. Cox proportional-hazards regression analysis was employed to compare the risk of death between the T2DM group and the T2DM–TB group.

**Results:**

A total of 101,837 potentially eligible patients were included in the study. After 1:10 propensity score matching, 1,258 patients were classified in the T2DM-TB group and 10,064 patients in the T2DM group. After adjustment for age, sex and comorbidities, the T2DM–TB group showed a 2.16-fold higher mortality risk than the T2DM group (95% CI = 1.83–2.56, *p* < .001). The mortality risk remained higher after stratification by year. The log-rank test indicated that male sex, age ≥60 years, hypertension and heart failure were independent risk factors.

**Conclusions:**

TB increases mortality risk in patients with T2DM on long-term follow-up. The independent risk factors for mortality in patients with concurrent T2DM and TB disease include male sex, age ≥60 years, hypertension and heart failure.

**KEY MESSAGES**
- The co-presentation of T2DM and TB is an important emerging issue, especially in Asia.
- This study showed mortality risk was significantly higher in the T2DM–TB group compared with the T2DM group on long-term follow-up.
- Increased medical attention is necessary for patients with T2DM and a history of TB disease.

## Introduction

The “stop TB strategy” implemented by the World Health Organization (WHO) from 2006 to 2015 gradually decreased the incidence, prevalence, and mortality of tuberculosis (TB) [1]. The control and elimination of TB require intensive efforts to reduce vulnerability to the disease. Proximate risk factors for TB, such as HIV infection, malnutrition, diabetes, alcohol use, and smoking, increase vulnerability to TB infection by impairing the host’s defence [2]. The global prevalence of diabetes in adults has nearly doubled from 4.7% in 1980 to 8.5% in 2016 [2]. The immunosuppressive effects of type 2 diabetes mellitus (T2DM) increase susceptibility to infectious diseases, such as TB, and the increasing prevalence of T2DM impairs the prevention and control of TB infection.

Both TB and diabetes are endemic in many regions of the world, and studies have revealed an association between the two diseases. The meta-analyses conducted by Jeon et al. [3] and Al-Rifai et al. [4] indicated T2DM was associated with a 3- to 3.6-fold increase in TB risk. Poor glycaemic control is also known as a risk factor for TB infection. Lee et al. found that TB infection risk in patients with diabetes was twofold higher in those with poor glycaemic control [fasting plasma glucose (FPG) >130 mg/dL] compared with those with good glycaemic control (FPG <130 mg/dL) and that the risk of TB infection did not differ significantly from that in individuals without diabetes [5]. Based on this, the WHO now considers T2DM an important emerging risk factor for TB infection. The risk of co-presentation of TB and T2DM is greater in Asia compared with those in other regions of the world [6,7]. A database search of T2DM studies conducted by Zheng et al. in developing Asian countries revealed that the prevalence of T2DM in patients with TB ranged from approximately 5% to >50%, whereas the prevalence of TB was 1.8–9.5 times higher in patients with T2DM compared with that in the general population [8]. Currently, in Taiwan, the TB incidence rate has been declining in recent years overall [9], but the TB-DM prevalence is still an important issue due to the increasing number of T2DM and inadequate glycaemic control.

Studies have investigated the effects of T2DM on clinical presentation and treatment response in patients with TB [10–12]. However, data for long-term outcomes, mortality, and associated risk factors are scant and inconclusive. Previous studies have indicated that T2DM can adversely affect TB treatment outcomes [13–16], whereas others have reported that it does not [17–19]. Therefore, we used the Taiwan National Health Insurance Research Database (NHIRD) to analyse the mortality and related risk factors in the co-presentation of active TB disease and T2DM among the nationwide population of Taiwan.

## Materials and methods

### Data sources

The Taiwan National Health Insurance Program is a universal healthcare system that covers 99% of the national population (23.3 million people) and has one of the largest population-based healthcare databases in the world. The NHIRD contains encrypted patient identification numbers and International Classification of Diseases, Ninth Revision, Clinical Modification (ICD-9-CM) codes for applied clinical diagnoses and procedures, details of prescribed drugs, dates of admission and discharge, and basic sociodemographic information, including sex and date of birth. Data were obtained from the Longitudinal Cohort of Diabetes Patients (LHDB), a sub-dataset of the NHIRD that contains randomised selected data (120,000 patients per year) from patients newly diagnosed with T2DM. The LHDB defines an inpatient with T2DM as a patient who has been diagnosed with T2DM (ICD-9-CM 250) on at least one visit or prescribed antidiabetic medication. An ambulatory patient with T2DM is defined as a patient who has been diagnosed with T2DM on at least two different visits or diagnosed with T2DM on at least one visit and has been prescribed antidiabetic medication.

### Design

**Figure 1.**
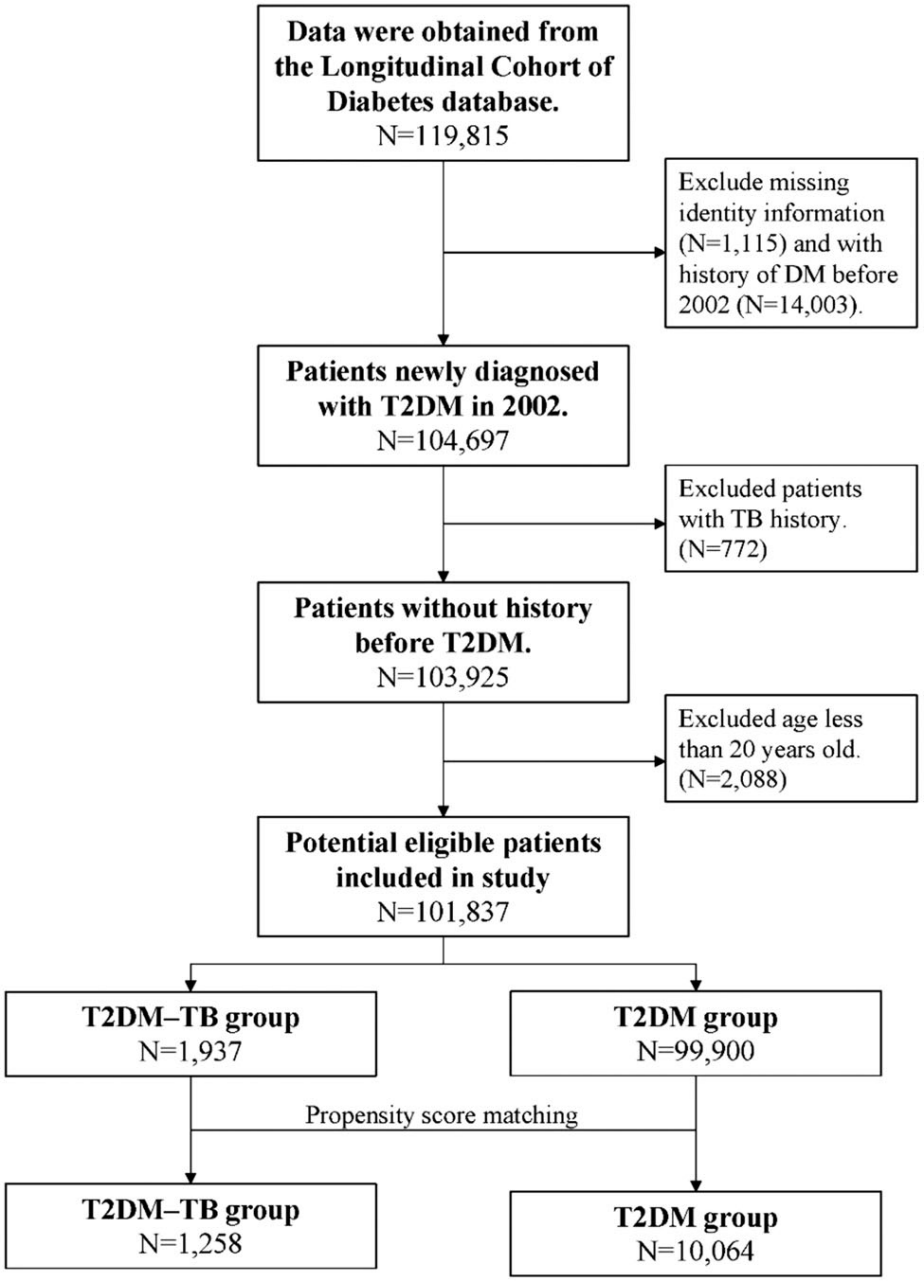
Flow chart of the patient identification and selection process.

This was a longitudinal retrospective cohort study that enrolled ambulatory patients aged >20 years from the LHDB who were first diagnosed with T2DM (ICD9-CM 250) during the period from 1 January 2002, to 31 December 2002. Among these patients, those who also had new diagnostic codes for TB (ICD-9-DM code 010.0–018.96) and had been prescribed anti-TB medications for more than 28 days were defined as the DM–TB group. Patients with a new diagnosis of TB before T2DM were excluded. The control group (T2DM group) comprised patients who were diagnosed with T2DM but not TB. For each patient in the T2DM–TB group, eight controls were randomly selected from the Longitudinal Health Insurance Database 2000. In the T2DM–TB group, the index date was taken as the date of the first diagnosis of TB according to the database. In the T2DM group, the index date was taken as the date of the first diagnosis of TB of the matched pair. In both groups, the data for patients who had died between the T2DM diagnosis and TB index dates were deleted. Propensity score matching was applied to match the T2DM and the T2DM–TB groups by sex, age at T2DM diagnosis, and selected comorbidities. Propensity scores were calculated using a logistic regression model that included the dependent variable. The model estimated the odds of TB diagnosis, and the aforementioned confounding variables were employed as independent variables. Next, the SAS matching macro function of “%OneToManyMTCH” proposed in the proceedings of the 29th SAS Users Group International was used. Baseline comorbidities affecting mortality that may have presented prior to the index date were defined as follows: hypertension (ICD-9 codes 401–405), coronary artery disease (ICD-9 codes 410–414), cardiovascular disease (ICD-9 codes 410–414), chronic obstructive pulmonary disease (ICD-9 codes 490–496, 500–505, 5064), and heart failure (ICD-9 codes 428). Comorbid conditions were recorded if they had occurred in either an inpatient setting or in three or more ambulatory care claims coded 12 months prior to the index medical care date. Hazard ratios (HRs) were adjusted by age group, sex, and comorbidities. A flowchart of the patient enrolment process is presented in Figure 1.

### Ethics statement

The study protocol has been approved and granted exemption from review by the Institutional Review Board of Chi Mei Medical Centre (Applicant’s No.: 10903-E04).

### Definition of mortality

Patients were followed up from the index date until the end of the database period (31 December 2011) or until the date of death, whichever occurred first. All citizens and legal residents of Taiwan were legally required to enrol in the NHI healthcare system. After death, the residents were required to be registered within 30 days at the district office and immediately withdrawn from the NHI. Therefore, patients were presumed dead if their inpatient claims indicated that they had been withdrawn from NHI enrolment within 30 days after discharge from their last hospitalization, and the discharge date was designated as the date of death.

### Statistical analysis

The significance of differences in baseline characteristics and comorbid variables between the two cohorts was evaluated by Student’s *t*-test for continuous variables and Pearson’s χ2 test for categorical variables. The standardised difference was calculated to compare the distributions of baseline covariates between the two groups in order to verify the balance between the two groups. A standardised difference of ≥0.1 was considered indicative of imbalance. Kaplan–Meier analysis was applied to calculate the differences in cumulative survival rates between different groups in the two cohorts, and log-rank test was applied to analyse the differences between survival curves. Cox proportional-hazards regression analysis was employed to compare the risk of death between the T2DM group and the T2DM–TB groups after adjusting for possible confounding factors. All statistical analyses were conducted using the SAS software for Windows 9.4 (SAS Institute, Inc., Cary, NC, USA), and *p* > .05 were considered statistically significant.

## Results

Table 1 compares the demographic characteristics and comorbid disorders between the T2DM–TB group (*n* = 1,258) and the propensity score-matched T2DM group (*n* = 10,064). The mean age was 59.17 ± 13.84 years in the T2DM–TB group and 59.32 ± 13.72 years in the T2DM group. There were no significant differences in baseline comorbidities between the two groups.

**Table 1.** Baseline demographic characteristics and comorbidities for patients with type 2 diabetes mellitus and tuberculosis (T2DM–TB group) and matched type 2 diabetes mellitus patients without TB (T2DM group).

	T2DM–TB group (*n* = 1258)	T2DM group (*n* = 10,064)	*p-*value	Standardised difference
Characteristic	*n* (%)	*n* (%)		
Age (mean ± SD)	59.17 ± 13.84	59.32 ± 13.72	.7126	−.01
Age group			.8607	
20–40	107 (.09)	811 (.08)		.02
40–60	519 (.41)	4172 (.41)		.00
≥60	632 (.5)	5081 (.5)		.00
Sex			.7948	
Male	880 (.7)	7004 (.7)		.01
Female	378 (.3)	3060 (.3)		−.01
Baseline comorbidity				
HTN	291 (.23)	2348 (.23)	.8751	.00
CAD	96 (.08)	750 (.07)	.8201	.01
COPD	33 (.03)	212 (.02)	.2350	.03
HF	29 (.02)	238 (.02)	.8955	.00
CVD	84 (.07)	586 (.06)	.2259	.04

CAD: coronary artery disease; COPD: chronic obstructive pulmonary disease; CVD: cerebrovascular disease; HF: heart failure; HTN: hypertension; T2DM: type 2 diabetes mellitus; TB: tuberculosis.

The incidence rate of mortality of two groups is shown in Table 2. The T2DM-TB group had a higher incidence rate of mortality in various age subgroups than T2DM group. The highest incidence of mortality in these T2DM patients with or without TB was over 65 years of age (33.5 and 19.1 per 1000 person-years, respectively).

**Table 2.** Incidence rate of mortality of T2DM-TB group and T2DM group stratified by patient age.

Age, years	T2DM–TB group			T2DM group		
	Number of death	Person year	Incidence rate^a^	Number of death	Person year	Incidence rate^a^
20–24	0	29.7	.0	0	230.3	.0
25–34	8	220.5	36.3	5	1879.5	2.7
35–44	14	811.2	17.3	20	6539.8	3.1
45–54	24	1562.2	15.4	59	12947.3	4.6
55–64	28	1532.8	18.3	110	12777.7	8.6
≥65	96	2868.4	33.5	464	24276.8	19.1

^a^Per 1000 person-years.

Table 3 presents the crude hazard ratios (HR) and adjusted hazard ratio (AHR) for all-cause mortality during the follow-up period. After adjusting for selected comorbid conditions, the T2DM–TB group exhibited a significantly higher risk of mortality compared with the T2DM group (AHR, 2.16; 95% CI, 1.83–2.56; *p* < .001). In the T2DM–TB group, mortality risk was significantly higher in patients aged ≥60 years compared with those aged 20–40 years (AHR, 3.03; 95% CI, 2.02–4.53, *p* < .001). Additionally, hypertension and heart failure were a significant risk factors for long-term mortality in the T2DM–TB group (AHR, hypertension: 1.29; 95% CI, 1.09–1.52; AHR, heart failure: 1.41; 95% CI, 1.02–1.94; *p* < .001).

**Table 3.** Hazard ratios and 95% confidence intervals of Cox proportional-hazards regression analysis during the follow-up of the study cohort.

Characteristics	Crude HR (95% CI)	*p*-value	Adjusted HR^a^ (95% CI)	*p*-value	Adjusted HR^b^ (95% CI)	*p*-value
TB						
Yes	2.16 (1.82–2.56)	<.001	2.16 (1.83–2.56)	<.001	9.94 (4.51–21.89)	<.001
No	Ref.		Ref.		Ref.	
Age group (years)						
20–40	Ref.		Ref.		Ref.	
40–60	1.22 (.80–1.85)	.358	1.14 (.75–1.74)	.528	1.91 (1.03–3.54)	.039
≥60	3.41 (2.29–5.09)	<.001	3.03 (2.02–4.53)	<.001	5.57 (3.06–1.15)	<.001
TB and age group						
With TB and age in 40–60	.28 (.12–.67)	.004	–		.29 (.12–.68)	.005
Without TB and age in 40–60	Ref.		–		Ref.	
With TB and age ≥60	.18 (.08–.41)	<.001	–		.18 (.08–.41)	<.001
Without TB and age ≥60	Ref.		–		Ref.	
Gender						
Male	1.44 (1.22–1.70)	<.001	1.56 (1.33–1.85)	<.001	1.57 (1.33–1.85)	<.001
Female	Ref.		Ref.		Ref.	
Comorbidity						
HTN	1.76 (1.52–2.03)	<.001	1.29 (1.09–1.52)	.002	1.29 (1.10–1.52)	.002
CAD	1.64 (1.34–2.01)	<.001	1.10 (.88–1.38)	.410	1.10 (.87–1.38)	.422
COPD	2.06 (1.50–2.83)	<.001	1.15 (.83–1.60)	.409	1.16 (.83–1.61)	.382
HF	2.11 (1.55–2.88)	<.001	1.41 (1.02–1.94)	.040	1.39 (1.00–1.92)	.047
CVD	1.74 (1.40–2.18)	<.001	1.13 (.90–1.43)	.299	1.14 (.90–1.43)	.285
LR test^c^, *p*-value			<.001			

CAD: coronary artery disease; CI: confidence interval; COPD: chronic obstructive pulmonary disease; CVD: cerebrovascular disease; HF: heart failure; HR: hazard ratio; HTN: hypertension; LR test: likelihood ratio test; T2DM: type 2 diabetes mellitus; TB: tuberculosis.

^a^Adjusted for age group, sex, and comorbidity.

^b^Adjusted for sex and comorbidity and included interaction term with age group.

^c^Likelihood ratio test for the full model, included interaction term of one characteristic and adjusted for others, and the nested model without interaction term.

**Figure 2.**
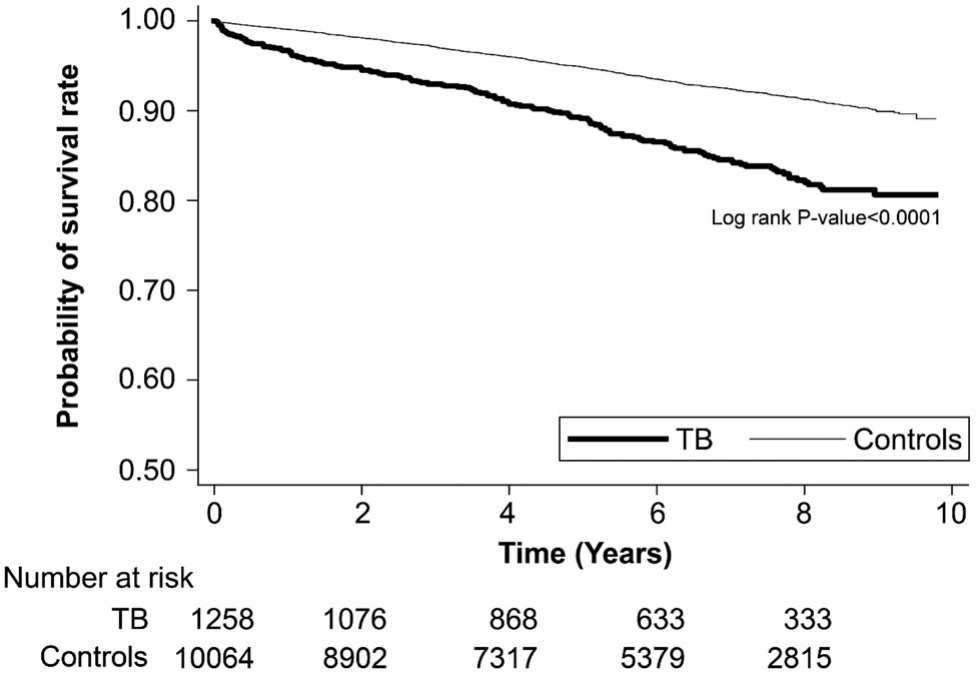
Kaplan–Meier survival analyses revealed that probability of survival was significantly lower in the T2DM–TB group compared with the T2DM group (control) (*p* < .001, log-rank test).

Table 4 stratifies the risk of death in all patients by years of follow-up. Mortality risk was significantly higher in the T2DM–TB group compared with that in the T2DM group during the first year of follow-up (HR, 3.47; 95% CI, 2.41–5.01; *p* < .0001), 1–2 years of follow-up (HR, 2.31; 95% CI, 1.47–3.63), 2–4 years of follow-up (HR, 1.91; 95% CI, 1.36–2.69), and ≥4 years of follow-up (HR, 1.95; 95% CI, 1.49–2.55). Figure 2 presents the results of the Kaplan–Meier analysis, which revealed a significantly lower survival rate in the T2DM–TB group compared with the T2DM group (*p* < .001, log-rank test).

**Table 4.** Hazard ratios and 95% confidence values for death in Cox proportional-hazards regression analysis stratified by year of follow-up in the study cohort.

Follow-up period	T2DM–TB group		T2DM group			
	*n*	Deaths	*n*	Deaths	HR (95% CI)	*p*-value
0–1 year	1258	41	10,064	96	3.47 (2.41–5.01)	<.0001
1–2 years	1162	24	9499	86	2.31 (1.47–3.63)	.0003
2–4 years	1076	40	8902	178	1.91 (1.36–2.69)	.0002
≥4 years	868	65	7317	298	1.95 (1.49–2.55)	<.0001

HR: hazard ratio; T2DM: type 2 diabetes mellitus; TB: tuberculosis.

## Discussion

This is the largest population-based cohort study investigating long-term outcomes in the co-presentation of tuberculosis and T2DM. After adjusting for selected comorbid conditions, mortality risk was 2.16 times higher in the T2DM–TB group than in the T2DM group. Age >60 years, male sex, hypertension, and heart failure were associated with an increased risk of death in this study.

Our finding is consistent with previous cohort studies involving the co-presentation of DM with other infectious diseases. Kim et al. [20] found that patients with diabetes in South Korea had a greater risk of infection-related hospitalizations and deaths after a 9-year follow-up. An epidemiological study in Italy showed that patients with diabetes had a significantly high mortality from infectious diseases [21]. In Taiwan, Ko et al. [22] use NHIRD to investigate the effect of DM on all-cause mortality among patients with newly diagnosed TB in Taiwan during 2000–2010. The mortality rate of TB-DM cohort was 26.1/100 person-years and DM was an independent risk factor for all-cause mortality from their study. Differently, their research group is TB patient with and without DM and our study is all DM patient. Additionally, our study results indicate that the mortality rate is persistently higher from 1 year to more than 4 years’ follow-up. This observation is compatible with a previous TB cohort study on the general population. A 5-year, population-based longitudinal study in Brazil showed patients with TB were at a higher risk of death even after treatment [23]. In a short period after TB infection, a higher mortality rate was correlated with delayed diagnosis, lower concentrations of anti-TB drugs, and a weak innate immune response [24]. However, after a long-term follow-up of over 4 years in this cohort study, the mortality risk remained higher in the matched DM group. Some speculations have been made based on observation of the general population with a TB infection. First, patients with TB frequently develop pulmonary sequelae, even after successful treatment [25]. Second, some studies suggest that TB is associated with cardiovascular disease. Chung et al. [26] reported a 40% increased incidence of acute coronary syndrome in patients with TB. A review by Huaman et al. [27] summarised hypothesised biological mechanisms of cerebrovascular disease in people with TB. Third, TB is also associated with increased cancer risk, especially lung cancer [28]. Whether we could apply these observations to the TB–T2DM population is uncertain.

In this long-term cohort study, we found that male sex, age >60 years, hypertension, and heart failure are independent factors for mortality risk. In one observation study of TB in Taiwan, Feng et al. [29] pointed out that male sex is associated with worse treatment outcomes due to a higher likelihood of smoking and more comorbidities. They suggested that sex-specific strategies, such as smoking cessation, are warranted to optimise TB management. Additionally, the nationwide, population-based cohort study by Chung et al. [26] that investigated the risk factors for acute coronary syndrome in patients with TB also identified male sex, age, hypertension, and diabetes as independent factors. Although the actual mechanisms contributing to the mortality risk in TB-DM comorbidity remain unknown, cardiovascular disease might play a substantial role in this relationship. Further studies are required to investigate the cause of death after long-term follow-up.

The strength of this study is the size of the dataset. This is the largest cohort study investigating long-term outcomes of TB in a population of patients with T2DM. In addition, most previous studies that investigated the effects of TB on T2DM treatment outcomes have either compared TB patients with T2DM with TB patients without T2DM or compared subgroups of patients with TB disease. This study compared patients with concurrent T2DM and TB with patients with T2DM without TB, with a view of determining the long-term consequences of TB infection in a population with T2DM, including patients fully treated for TB. Our findings confirm and extend the existing knowledge of the influence of TB infection on T2DM, and the finding on increased long-term mortality risk is novel.

Some limitations of this study need to be considered. First, ICD-9-CM codes were used to identify various diseases among the study population, and some diseases may have been misclassified. To maximise the accuracy of identifying TB patients, only those with appropriate ICD-9-CM codes and those that had been prescribed more than two anti-TB medications for more than 28 days were recruited. A second limitation is that, since TB disease may not be clinically evident, some of the patients included in the T2DM group may have had undiagnosed TB. Third, the patient database did not include characteristics, such as glycaemic control, environmental factors, socioeconomic factors, and use of tobacco or alcohol. Although propensity score matching helped reduce the selection bias, it only addressed observable variables, and unobservable variables may still have had confounding effects on our results. Fourth, details about the cause of death were not available in the database. Future studies should be designed to collect detailed information about the cause of death.

In conclusion, our study found that active TB increased the risk of death in a T2DM population. Risk of death significantly increased with follow-up duration. Mortality risk factors in patients with T2DM included old age (>60 years), male sex, hypertension, and heart failure. Therefore, care providers specialising in diabetes should pay close attention to these high-risk patients for the first year after diagnosis and well into the succeeding decades. Examples of management strategies include sending these patients for lung assessment and rehabilitation, ensuring that they consistently avoid smoking, and reducing cardiovascular risk as much as possible.

## Data Availability

The data that support the findings of this study are available from the corresponding author (Kai-Jen Tien, e-mail: tkzzkimo@gmail.com) upon reasonable request.
